# Unpacking “OK, Boomer”: Using Lifespan Concepts to Ease Intergenerational Conflict

**DOI:** 10.1093/geroni/igaa057.1713

**Published:** 2020-12-16

**Authors:** Allyson Graf, Robin Bartlett

**Affiliations:** Northern Kentucky University, Highland Heights, Kentucky, United States

## Abstract

With the “OK, Boomer” media exchange in late 2019, intergenerational conflict is touted as existing at an all-time high. Although the age diversity of today’s workforce is unprecedented, spanning nearly five generations of workers, generational stereotyping and its influence on the identities and experiences of those individuals is not new. In this talk, we will advocate for the role that lifespan developmental psychology can play in preparing students to enter a sometimes contentious, misrepresented multigenerational workforce. We will demonstrate the value of helping students distinguish normative age-graded, normative history-graded, and non-normative influences to better understand individual similarities and differences in developmental experiences. We will discuss research on age identity and generational identity as distinct and self-enhancing life-span processes. Finally, we will highlight the developmental barriers that must be navigated in order to foster intergenerational cohesion.

